# Proteomics of Crystal–Cell Interactions: A Model for Kidney Stone Research

**DOI:** 10.3390/cells8091076

**Published:** 2019-09-12

**Authors:** Visith Thongboonkerd

**Affiliations:** Medical Proteomics Unit, Office for Research and Development, Faculty of Medicine Siriraj Hospital, Mahidol University, Bangkok 10700, Thailand; thongboonkerd@dr.com or vthongbo@yahoo.com; Tel.: +66-24-192-850

**Keywords:** CaOx, COD, COM, exosome, mass spectrometry, nephrolithiasis, secretome, urolithiasis

## Abstract

Nephrolithiasis/urolithiasis (i.e., kidney stone disease) remains a global public health problem with increasing incidence/prevalence. The most common chemical composition of kidney stones is calcium oxalate that initiates stone formation by crystallization, crystal growth, crystal aggregation, crystal–cell adhesion, and crystal invasion through extracellular matrix in renal interstitium. Among these processes, crystal–cell interactions (defined as “the phenomena in which the cell is altered by any means of effects from the crystal that adheres onto cellular surface or is internalized into the cell, accompanying with changes of the crystal, e.g., growth, adhesive capability, degradation, etc., induced by the cell”) are very important for crystal retention in the kidney. During the past 12 years, proteomics has been extensively applied to kidney stone research aiming for better understanding of the pathogenic mechanisms of kidney stone formation. This article provides an overview of the current knowledge in this field and summarizes the data obtained from all the studies that applied proteomics to the investigations of crystal–cell interactions that subsequently led to functional studies to address the significant impact or functional roles of the expression proteomics data in the pathogenesis of kidney stone disease.

## 1. Introduction

Kidney stone disease remains a common human disease and can be found in both developed and developing countries around the globe [1,2,3]. Kidney stones comprise mainly calcium-containing crystals, particularly calcium oxalate (CaOx) monohydrate (COM) and CaOx dihydrate (COD) [1,2,3]. Mechanistic processes for kidney stone formation are quite sophisticated involving crystallization, crystal growth, crystal aggregation, crystal–cell adhesion, and crystal invasion through extracellular matrix (ECM) in renal interstitium [4,5,6]. Crystallization can occur either inside renal tubules (intratubular model) or at the renal interstitium (Randall’s plaque model) [4,5,6]. After the crystals are formed, individual crystals become the larger particles by either crystal growth or aggregation mechanism [7,8]. In addition, crystal–cell adhesion causes crystal retention inside the renal tubules or interstitium [9,10]. The adhered crystals can be internalized into renal tubular cells for degradation or, vice versa, further enhancement of the stone formation process via inflammatory cascade [11,12]. Finally, the internalized crystals or crystals formed in the renal interstitium can invade or migrate to other locales through the ECM using the plasmin–plasminogen pathway [13] and subsequently trigger tissue inflammation and erosion [14,15].

Interestingly, one of the crucial processes for crystal retention and stone formation is the crystal–cell adhesion step that requires “crystal–cell interactions” which can be defined herein as the phenomena in which the cell is altered by any means of effects from the crystal that adheres onto cellular surface or is internalized into the cell, accompanying with changes of the crystal, e.g., growth, adhesive capability, degradation, etc., induced by the cell. Using the term “interactions” is logical by means of “reciprocal actions” between the crystal and the cell. It is obvious that the crystal can cause many changes in the cell, from mild to severe cytotoxicities [14,15,16]. On the other hand, composition of the cell, especially on the apical surface and in endocytic vesicle, can affect growth, adhesive capability, and degradation of the crystal [17,18]. Such interactions can enhance intrarenal crystal retention and endocytosis of the crystals into renal tubular cells. Moreover, crystal–cell interactions can also lead to renal tubular cell injury and inflammatory cascade that further enhance the stone formation process [14,15,16].

During the proteomics era, proteomics has been widely applied to various kidney diseases [19,20,21]. Within the past 12 years, proteomics has been extensively applied to the investigations of kidney stone disease, particularly COM and COD types, aiming for better understanding of the pathogenic mechanisms of kidney stone formation [22,23]. This article summarizes all the studies that applied proteomics to the investigations of crystal–cell interactions that subsequently led to functional studies to address the significant impact or functional roles of the expression proteomics data in the pathogenesis of kidney stone disease. Note that the studies, which applied proteomics to identify proteins in the urine or kidney stone matrices from the stone formers without any evidence for “crystal–cell interactions” (see definition above), were excluded from this review (because many of those proteins were simply mixed with stone modulators in the urine or just entrapped inside the stone matrices by stagnation during the stone enlargement without any role in the stone pathogenesis). All of the relevant studies in kidney stone research related to proteomics of CaOx crystal–cell interactions are summarized in Table 1 and discussed as follows.

## 2. Effects of Differential Doses and Types of CaOx Crystals on Cellular Proteome of Renal Tubular Cells

The very first study of proteomics applied to crystal–cell interactions was done in 2008 [24]. In this work, the study model was carefully established showing that COM crystals at a dosage of 100 µg/mL (per volume of culture medium) with an incubation period at 48 h did not cause severe cytotoxicity to renal tubular cells and percentage of cell death was not increased. Changes in cellular proteome induced by COM crystals thus reflected response of the cells to the COM crystals, not their cytotoxic effects. Moreover, scanning electron microscopy (SEM) showed distortion of the COM crystal borders after incubating with the cells, indicating the reciprocal crystal–cell interactions for the first time with direct experimental evidence. Using two-dimensional gel electrophoresis (2-DE) with Sypro Ruby fluorescence staining followed by quadrupole time-of-flight (Q-TOF) mass spectrometry (MS) and tandem MS (MS/MS), 11 upregulated and five downregulated proteins that play roles in transcription/translation, signal transduction, cellular metabolism and growth, nuclear and cellular structure, transport, stress response, and biosynthesis were identified. Some of these proteomics data were confirmed by two-dimensional (2-D) Western blotting [24]. 

Subsequently, a subcellular proteome study [29] was performed to evaluate effects of 100 µg/mL COM crystals on mitochondrial proteome and function in renal tubular cells exposed to the crystals for 48 h using a highly purified mitochondrial isolation method [47]. Comparative analysis using 2-DE with Deep Purple fluorescence staining followed by Q-TOF MS and MS/MS analyses identified 12 upregulated and three downregulated mitochondrial proteins that regulate cell structure, metabolism, and energy production. Additionally, the Oxyblot assay demonstrated that COM crystals induced mitochondrial dysfunction and accumulation of oxidatively modified proteins in the cells [29]. 

Another study using a higher dose (200 µg/mL) of COM crystals revealed that cell death was not increased when renal tubular cells were incubated with COM crystals and 2 mM oxalate for a shorter period (only 12 h) [27]. Using 2-DE with silver staining followed by liquid chromatography coupled with electrospray ionization MS/MS (LC-ESI MS/MS), the data showed nine upregulated and three downregulated cellular proteins with roles in energy production, cell proliferation, apoptosis, stress response, calcium balance, and protein synthesis. 

With a much higher dose (1000 µg/mL) of COM crystals, the cells underwent severe cytotoxicity with increased total cell death after incubation with the crystals for 48 h [25]. Using 2-DE with Sypro Ruby staining followed by Q-TOF MS and MS/MS analyses, 25 upregulated and 23 downregulated proteins were identified. The decreased levels of ezrin, enolase-1, and annexin-A1 were confirmed by 2-D Western blotting. Interestingly, chaperones were upregulated, whereas other proteins involved in protein synthesis, cell cycle regulation, cell structure, and cellular signaling were downregulated [25]. Functional investigations to define the biological relevance of this expression proteomics dataset were recently performed using various assays [31]. Initially, all the significantly altered proteins were submitted to the STRING bioinformatic tool [48] to obtain the protein–protein interactions network, which revealed cell proliferation/wound healing, oxidative stress/mitochondrial function, and cellular junction complex/integrity as the main biological functions of the significantly altered proteins. Validation experiments showed that the cytotoxic renal tubular cells treated with 1000 µg/mL COM crystals had decreases in cell proliferation, wound-healing capability, trans-epithelial resistance (TER), and levels of tight junction protein zonula occludens-1 (ZO-1) and signaling protein RhoA. In contrast, levels of oxidatively modified proteins were increased. While the ATP synthase alpha subunit precursor was decreased, other mitochondrial proteins involved in energy generation and homeostasis (e.g., aconitase, adenylate kinase 2, ubiquinol-cytochrome-c reductase) were increased [25]. As a result, the intracellular ATP level was increased in the COM-treated cells [31]. These data might reflect the cellular processes that occurred during COM crystal-induced tubulotoxicity [31]. 

Proteomics was also applied to the investigations of changes in cellular proteome of renal tubular cells after exposure to the non-toxic dose (100 µg/mL) of COD crystals for 48 h [26]. Using 2-DE with Sypro Ruby staining followed by Q-TOF MS and MS/MS analyses, the data demonstrated five upregulated and five downregulated proteins that control cellular metabolism, structure, integrity, signal transduction, and stress response [26]. Subsequently, changes in glycoproteome of renal tubular cells induced by 100 µg/mL COD crystals were examined [28]. Using 2-DE and Pro-Q Emerald glycoprotein staining followed by normalization using Sypro Ruby total protein staining, 16 significantly altered glycoproteins, including glycoforms of three proteasome subunits responsible for regulation of cell–cell dissociation and actin-related protein 3 (ARP3) that played role in cellular integrity were identified by Q-TOF MS and MS/MS. These data suggest that COD crystals caused cell dissociation and reduced cellular integrity [28]. 

Furthermore, comparative analyses of functional perturbations of renal tubular cells after exposure to differential doses and types of CaOx crystals were performed [30]. Comparisons of the expression data revealed that the high-dose of these crystals caused more obvious changes in cellular proteome than the low-dose, and COM was more potent for inducing alterations in cellular proteome than COD. Functional analyses showed greater levels of intracellular ATP, oxidatively modified proteins, and cell death but lower levels of ubiquitinated proteins in the cells treated with high-dose COM and COD. The COM crystals also induced more severe cytotoxicity than COD. Pretreatment of the cells with epigallocatechin gallate (EGCG), an antioxidant, could lower the crystal-induced accumulation of oxidatively modified proteins and crystal-binding capability of the cells. These data highlighted the differential effects of a high-dose versus low-dose and COM versus COD crystals for inducing functional perturbations in renal tubular cells. The findings also demonstrated that oxidative stress might play a significant role in the crystal-binding capability of the cells treated with these differential doses/types of the crystals [30]. 

However, it should be noted that the dosage of COM/COD crystals and their exposure time to the cells applied in all the aforementioned in vitro studies might not precisely represent the in vivo condition in the stone formers that could be accompanied with another model of the kidney stone pathogenesis, i.e., Randall’s plaque model. Currently, the in vivo study of crystal–cell interactions seems not yet practical for kidney stone research by limitation (indeed lack) of technology to track crystal–cell interactions in vivo. When such technology becomes available, the pathogenic mechanisms of kidney stone formation will be much clearer.

## 3. Proteomic Identification of COM Crystal Receptors on Apical Surface of Renal Tubular Cells

Another step that makes a big leap in kidney stone research at the cellular level is the development of a simple but effective peeling method to isolate and purify apical membranes of the polarized renal tubular cells [49]. This novel method relies mainly on the adsorptive capability of an applied surface (e.g., Whatman filter paper, nitrocellulose membrane, cellophane or glass coverslip) to adhere with apical membranes using hydrous affinity and ionic interaction between such surfaces and apical membranes. Among these four surfaces tested, the filter paper was the most effective one to isolate and purify apical membranes without any contaminations from basolateral membrane proteins as confirmed by Western blotting and immunofluorescence staining [49]. Comparing to the conventional biochemical assays (mainly using centrifugation-based methods), the peeling method was much more effective to isolate/purify apical membranes. 

This peeling method was applied to isolate a highly purified fraction of apical membranes of polarized renal tubular cells followed by identification of potential COM crystal receptors on the cell surface [32]. The recovered proteins were resuspended in artificial urine and incubated overnight with COM crystals. After washing and elution, gel-enhanced liquid chromatography followed by tandem MS (GeLC-MS/MS) identified a total of 96 potential COM crystal receptors. Among these, the role for annexin II as the COM crystal receptor was validated by the crystal–cell adhesion assay followed by neutralization using a specific anti-annexin II antibody [32]. In addition, other COM crystal receptors were confirmed in subsequent studies, including α-enolase [34,36], annexin A1 [33,35], heat shock protein 90 (HSP90) [37,40], and plasma membrane Ca^2+^ ATPase 2 (PMCA2) [41]. 

Other proteins that did not serve as the COM crystal receptors but their roles were related to various COM crystal receptors included α-tubulin and lamin A/C. A-tubulin (a cytoskeletal protein) was identified as one of the downregulated proteins in renal tubular cells treated with 1000 µg/mL COM crystals using a proteomics approach [25] and served as the central node of a protein–protein interaction network [38]. Overexpression of α-tubulin led to downregulation of three COM crystal receptors, including α-enolase, heat shock protein 70 (HSP70), and HSP90, and declined crystal-binding capability of the cells [38]. In contrast, another proteomics study identified lamin A/C (a nuclear lamina protein) as one of the upregulated proteins in renal tubular cells treated with 100 µg/mL COM crystals [24] and served as the central node of a protein–protein interaction network [39]. Knockdown of lamin A/C by small interfering RNA (siRNA) caused reduced levels of four COM crystal receptors, including vimentin, α-enolase, S100, and annexin A2, and declined crystal-binding capability of the cells [39]. These data suggest that lamin A/C has various functions including regulation of the expression of other proteins. Characterizations of the aforementioned COM crystal receptors and their regulators or associated proteins may lead to further development of strategies to prevent kidney stone formation by blocking COM crystal adhesion to the cells and crystal retention inside the kidney.

## 4. Effects of COM Crystals on Cellular Proteome of Monocytes and Macrophages

The CaOx crystals interact with not only renal tubular cells but also monocytes and macrophages, which play important roles in inflammatory processes that further aggravate kidney stone formation. Therefore, the roles for monocytes/macrophages in response to COM crystals have been investigated by proteome studies. Human monocytic U937 cells were incubated with 100 µg/mL COM crystals for 24 h and changes in the cellular proteome were examined [42]. Using 2-DE with Deep Purple fluorescence staining followed by Q-TOF MS and MS/MS analyses, the findings showed increased levels of nine proteins and decreased levels of 13 proteins with roles in cell cycle, cellular structure, carbohydrate metabolism, lipid metabolism, mRNA processing, and protein synthesis, stabilization, and degradation [42]. 

Later, a similar approach was employed to investigate changes in the cellular proteome of human macrophages after interacting with 100 µg/mL COM crystals for 24 h [43]. Comparative proteome analysis revealed seven upregulated and seven downregulated proteins that control cellular structure, carbohydrate metabolism, DNA/RNA processing, protein metabolism, molecular trafficking, and stress response. Interestingly, protein–protein interaction network analysis showed that HSP90 directly interacted with β-actin and α-tubulin, and all of these interacting partners were upregulated in the COM-treated macrophages. Functional investigations revealed that only actin co-localized with HSP90 surrounding the engulfed COM crystal to form a phagosome structure. Knockdown of HSP90 by siRNA suppressed phagosome formation and phagocytic and migratory activities of macrophages induced by COM crystals. These data indicated that HSP90 not only served as the COM crystal receptor but also played a crucial role in phagosome formation and phagocytic activity of macrophages [43].

## 5. Effects of COM Crystals on Secretome and Exosomal Proteome

In addition to alterations of cellular proteome induced by COM crystals, the effects of crystal–cell interactions on secretome were also investigated using proteomics approaches. For renal tubular cells, 100 µg/mL COM crystals caused increased levels of two secretory proteins and decreased levels of four secretory proteins [13]. Interestingly, functional investigation was performed using a previously established crystal-migration assay based on plasminogen–plasmin activity [50]. The data showed that the increased secretory enolase-1, in turn, promoted COM crystal invasion and migration of monocytes through the ECM [13]. For human monocytes, incubating 100 µg/mL COM crystals with U937 monocytic cells for 16 h induced 14 increased and four decreased secretory proteins from the cells [44]. Interestingly, an immunofluorescence study detected increased HSP90 on the cell surface, implicating that its increased secretion might be from a non-classical secretory pathway [44]. 

To confirm that COM could induce changes in the secretome via a non-classical secretory pathway, subsequent studies investigated alterations in exosomal proteome derived from human macrophages. Using gel-based [45] and gel-free [46] quantitative proteomics approaches, a number of significantly altered exosomal proteins were identified. Interestingly, macrophages exposed to COM crystals produced greater levels of interleukin-1β (IL-1β), which is one of the inflammasome activation markers [51,52]. In addition, exosomes derived from these crystal-interacting macrophages induced migration/activation of monocytes that produced greater levels of interleukin-8 (IL-8) (a pro-inflammatory cytokine), which served as a paracrine to activate phagocytic activity of the macrophages [45]. Knockdown of one of the upregulated proteins (vimentin) by siRNA successfully suppressed such effects of the exosomes derived from COM crystal-exposed macrophages [45]. Moreover, exosomes derived from these crystal-interacting macrophages induced neutrophil migration, enhanced production of the pro-inflammatory cytokine IL-8 from renal tubular cells, had greater binding capability to COM crystals, and activated crystal invasion through ECM [46].

## 6. Conclusions

During the past 12 years, proteomics has been successfully applied to kidney stone research aiming for better understanding of the pathogenic mechanisms of kidney stone formation, particularly at the step of crystal–cell interactions (Table 1). The first phase of these studies dealt mainly with characterizations of the altered whole cell proteome in renal tubular cells after interacting with COM crystals. Subsequently, subcellular proteomics revealed additional information on the changes in mitochondrial proteome and mitochondrial dysfunction after the cells interacted with COM crystals. Additionally, functional investigations of the effects of differential doses and types of CaOx crystals on the cellular proteome of renal tubular cells have led to better understanding of the expression and functional perturbations of renal tubular cells affected by crystal–cell interactions (see Section 2). A big leap in this field occurred after a simple and highly effective method was developed to purify apical membranes from the polarized renal tubular cells. In particular, a large number of potential COM crystal receptors were identified by proteomics followed by functional validation in several subsequent studies. These findings are very important to further develop strategies to prevent kidney stone formation by blocking the binding between the causative crystals and the target renal tubular cells (see Section 3). In addition to renal tubular cells, proteomics has been also applied to the investigations of changes in the cellular proteome of human monocytes/macrophages that have led to a clearer picture of the mechanism to eliminate the adhered and internalized CaOx crystals and inflammatory response (see Section 4). Furthermore, several functional assays were developed to address functional significance of the expression data identified from the initial phase of proteomics studies. For example, identification of the altered secretory proteins induced by COM crystals that in turn aggravated crystal invasion through the ECM. Finally, the recent knowledge on the roles for exosomes derived from macrophages in inflammatory cascade of kidney stone formation has provided additional novel information to better understand the inflammatory cascade during crystal–cell interactions (see Section 5). Taken together, all of these findings have led to a better understanding of the pathogenic mechanisms of kidney stone disease, particularly at the step of crystal–cell interactions. Moreover, these findings may also lead to the development of novel strategies to prevent the formation of new and/or recurrent kidney stones.

## Figures and Tables

**Table 1:** Summary of all relevant studies related to proteomics of CaOx crystal–cell interactions.

Year	Authors/Reference	Conditions of Crystal–Cell Interactions	Proteomics Technologies/Functional Assays	Main Findings
**Effects of differential doses and types of CaOx crystals on cellular proteome of renal tubular cells**				
2008	Semangoen T, et al. [24]	Cell: MDCK cells (whole cells) Crystal: 100 µg/mL COM Incubation: 48 h	2-DE, Sypro Ruby staining, Q-TOF MS and MS/MS	11 upregulated and 5 downregulated proteins that play roles in transcription/translation, signal transduction, cellular metabolism and growth, nuclear and cellular structure, transport, stress response, and biosynthesis.
2008	Thongboonkerd V, et al. [25]	Cell: MDCK cells (whole cells) Crystal: 1000 µg/mL COM Incubation: 48 h	2-DE, Sypro Ruby staining, Q-TOF MS and MS/MS	25 upregulated and 23 downregulated proteins were identified. While chaperones were upregulated, other proteins involved in protein synthesis, cell cycle regulation, cell structure, and cellular signaling were downregulated.
2008	Semangoen T, et al. [26]	Cell: MDCK cells (whole cells) Crystal: 100 µg/mL COD Incubation: 48 h	2-DE, Sypro Ruby staining, Q-TOF MS and MS/MS	5 upregulated and 5 downregulated proteins that control cellular metabolism, structure, integrity, signal transduction, and stress response.
2010	Chen S, et al. [27]	Cell: HK-2 cells (whole cells) Crystal: 200 µg/mL COM Incubation: 12 h	2-DE, silver diamine staining, LC-ESI MS/MS	9 upregulated and 3 downregulated proteins with roles in energy production, cell proliferation, apoptosis, stress response, calcium balance, and protein synthesis.
2011	Chiangjong W, et al. [28]	Cell: MDCK cells (whole cells) Crystal: 100 µg/mL COD Incubation: 48 h	2-DE, Pro-Q Emerald glycoprotein staining, Sypro Ruby total protein staining, Q-TOF MS and MS/MS	Among 16 significantly altered glycoproteins, glycoforms of three proteasome subunits were upregulated, whereas a glycoform of actin-related protein 3 (ARP3) was downregulated.
2012	Chaiyarit S, et al. [29]	Cell: MDCK cells (purified mitochondria) Crystal: 100 µg/mL COM Incubation: 48 h	2-DE, Deep Purple staining, Q-TOF MS and MS/MS, Oxyblot assay	12 upregulated and 3 downregulated mitochondrial proteins that regulate cell structure, metabolism and energy production. COM crystals also induced mitochondrial dysfunction and accumulation of oxidatively modified proteins in the cells.
2017	Vinaiphat A, et al. [30]	Cell: MDCK cells (whole cells) Crystal: 100/1000 µg/mL COD/COM Incubation: 48 h	Protein–protein interactions network analysis, luciferin–luciferase ATP assay, Oxyblot assay, measurement of ubiquitinated proteins, cell death assay, and crystal–cell adhesion assay	High-dose of these crystals caused more obvious changes in cellular proteome than low-dose, and COM was more potent for inducing alterations in cellular proteome than COD. The cells treated with high-dose had greater levels of intracellular ATP, oxidatively modified proteins and cell death, but lower level of ubiquitinated proteins. COM also induced more severe cytotoxicity than COD. Pretreatment of the cells with an antioxidant EGCG could lower the crystal-induced accumulation of oxidatively modified proteins and crystal-binding capability of the cells.
2018	Peerapen P, et al. [31]	Cell: MDCK cells (whole cells) Crystal: 1000 µg/mL COM Incubation: 48 h	Protein–protein interactions network analysis, proliferation assay, wound-healing assay, Oxyblot assay, luciferin–luciferase ATP assay, and measurement of trans-epithelial resistance (TER)	The cytotoxic cells treated with 1000 µg/mL COM crystals had decreases in cell proliferation, wound-healing capability, TER, and levels of tight junction protein zonula occludens-1 (ZO-1) and signaling protein RhoA. In contrast, levels of intracellular ATP and oxidatively modified proteins were increased.
**Proteomic identification of COM crystal receptors on apical surface of renal tubular cells**				
2011	Fong-ngern K, et al. [32]	Cell: MDCK cells (purified apical membranes) Crystal: COM Incubation: overnight	GeLC-MS/MS, Q-TOF MS and MS/MS, crystal–cell adhesion assay, antibody neutralization assay	A total of 96 potential COM crystal receptors were identified. The role for annexin II as the COM crystal receptor was confirmed by neutralization assay using a specific antibody.
2012	Chutipongtanate S, et al. [33]	Cell: MDCK cells (whole cells and purified apical membranes) Crystal: 100 µg/mL COM Incubation: 30 min, 72 h	2-DE, Sypro Ruby staining, Q-TOF MS and MS/MS, crystal–cell adhesion assay, calcium induction assay, antibody neutralization assay	Annexin A1 served as the COM crystal receptor.
2013	Kanlaya R, et al. [34]	Cell: MDCK cells (whole cells and purified apical membranes) Crystal: 100 µg/mL COM Incubation: 30 min, 72 h	2-DE, Sypro Ruby staining, Q-TOF MS and MS/MS, crystal–cell adhesion assay, oxalate induction assay, antibody neutralization assay	A-enolase served as the COM crystal receptor.
2016	Peerapen P, et al. [35]	Cell: MDCK cells (whole cells and purified apical membranes) Crystal: 100 µg/mL COM Incubation: 30 min	Crystal–cell adhesion assay, calcium induction assay	Annexin A1 served as the COM crystal receptor.
2016	Fong-ngern K, et al. [36]	Cell: MDCK cells (whole cells and purified apical membranes) Crystal: 100 µg/mL COM Incubation: 1 h	Crystal–cell adhesion assay, antibody neutralization assay	A-enolase served as the COM crystal receptor.
2016	Fong-ngern K, et al. [37]	Cell: MDCK cells (whole cells and purified apical membranes) Crystal: 100 µg/mL COM Incubation: 1 h, 48 h	Crystal–cell adhesion assay, crystal internalization assay, antibody neutralization assay, protein knockdown by small interfering RNA (siRNA)	HSP90 served as the COM crystal receptor.
2016	Manissorn J, et al. [38]	Cell: MDCK cells (whole cells and purified apical membranes) Crystal: 100 µg/mL COM Incubation: 30 min	Crystal–cell adhesion assay, protein overexpression, oxalate induction assay	A-tubulin overexpression caused downregulation of three COM crystal receptors, including α-enolase, HSP70 and HSP90, and declined crystal-binding capability of the cells.
2016	Pongsakul N, et al. [39]	Cell: MDCK cells (whole cells) Crystal: 100 µg/mL COM Incubation: 30 min	Crystal–cell adhesion assay, protein knockdown by siRNA, oxalate induction assay	Lamin A/C knockdown caused reduced levels of four COM crystal receptors, including vimentin, α-enolase, S100 and annexin A2, and declined crystal-binding capability of the cells.
2018	Manissorn J, et al. [40]	Cell: HEK293T cells (whole cells) Crystal: 100 µg/mL COM Incubation: 1 h	Crystal–cell adhesion assay, protein knockdown by siRNA	HSP90 served as the COM crystal receptor.
2018	Vinaiphat A, et al. [41]	Cell: MDCK cells (whole cells and purified apical membranes) Crystal: 100 µg/mL COM Incubation: 1 h	Crystal–cell adhesion assay, crystal internalization assay, antibody neutralization assay	PMCA2 served as the COM crystal receptor.
**Effects of COM crystals on cellular proteome of monocytes and macrophages**				
2010	Singhto N, et al. [42]	Cell: U937 cells (whole cells) Crystal: 100 µg/mL COM Incubation: 24 h	2-DE, Deep Purple staining, Q-TOF MS and MS/MS	9 upregulated proteins and 13 downregulated proteins with roles in cell cycle, cellular structure, carbohydrate metabolism, lipid metabolism, mRNA processing, and protein synthesis, stabilization and degradation.
2013	Singhto N, et al. [43]	Cell: Human macrophages (whole cells) Crystal: 100 µg/mL COM Incubation: 24 h	2-DE, Deep Purple staining, Q-TOF MS and MS/MS, phagocytosis assay, protein knockdown by siRNA	7 upregulated and 7 downregulated proteins that control cellular structure, carbohydrate metabolism, DNA/RNA processing, protein metabolism, molecular trafficking, and stress response. HSP90 played crucial role in phagosome formation and phagocytic activity of macrophages.
**Effects of COM crystals on secretome and exosomal proteome**				
2016	Chiangjong W, et al. [13]	Cell: MDCK cells (secretome) Crystal: 100 µg/mL COM Incubation: 20 h	2-DE, Deep Purple staining, Q-TOF MS/MS, crystal-migration assay, cell-migration assay	COM crystals induced 2 increased and 4 decreased secretory proteins from renal tubular cells. The increased secretory enolase-1, in turn, enhanced COM crystal invasion through ECM.
2016	Sintiprungrat K, et al. [44]	Cell: U937 cells (secretome) Crystal: 100 µg/mL COM Incubation: 16 h	2-DE, Deep Purple staining, Q-TOF MS and MS/MS	COM crystals induced 14 increased and 4 decreased secretory proteins associated with immune response and cell survival from U937 monocytic cells. The increased HSP90 was localized on the cell surface.
2018	Singhto N, et al. [45]	Cell: Human macrophages (exosomes) Crystal: 100 µg/mL COM Incubation: 24 h	2-DE, Deep Purple staining, nanoLC-ESI-ETD MS/MS, cell-migration assay, phagocytosis assay, protein knockdown by siRNA	2 upregulated and 4 downregulated exosomal proteins. Exosomes derived from the crystal-interacting macrophages induced migration/activation of monocytes and activated phagocytic activity of the macrophages. Knockdown of vimentin by siRNA successfully suppressed such effects of the exosomes derived from COM crystal-exposed macrophages.
2018	Singhto N, et al. [46]	Cell: Human macrophages (exosomes) Crystal: 100 µg/mL COM Incubation: 24 h	nanoLC-ESI-Qq-TOF, cell-migration assay, crystal-binding assay, crystal-migration assay	7 upregulated and 19 downregulated exosomal proteins. Exosomes derived from the crystal-interacting macrophages induced neutrophil migration, enhanced production of a pro-inflammatory cytokine IL-8 from renal tubular cells, had greater binding capability to COM crystals, and activated crystal invasion through ECM.
